# Supplementary figures and images for: ESAT-6 undergoes self-association at phagosomal pH and an ESAT-6 specific nanobody restricts M. tuberculosis growth in macrophages
Source: bioRxiv. 2024 Mar 19:2023.08.16.553641. Originally published 2023 Aug 17. Preprint. [Version 3] doi: 10.1101/2023.08.16.553641 (PMC10462100; doi:10.1101/2023.08.16.553641)

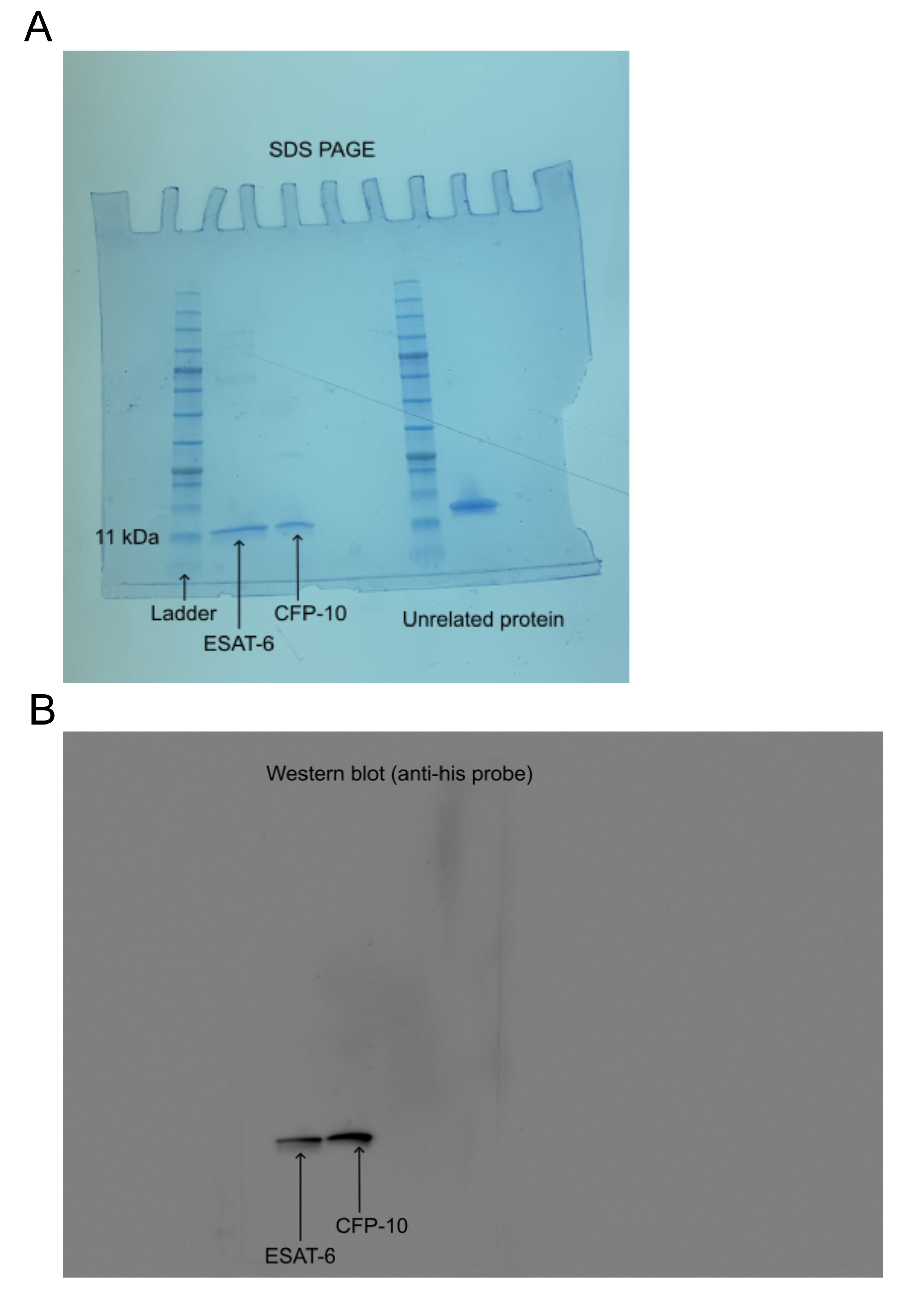

Supplement: Supplement 1 [file media-1.zip › Figure 1 - source data 1.png]

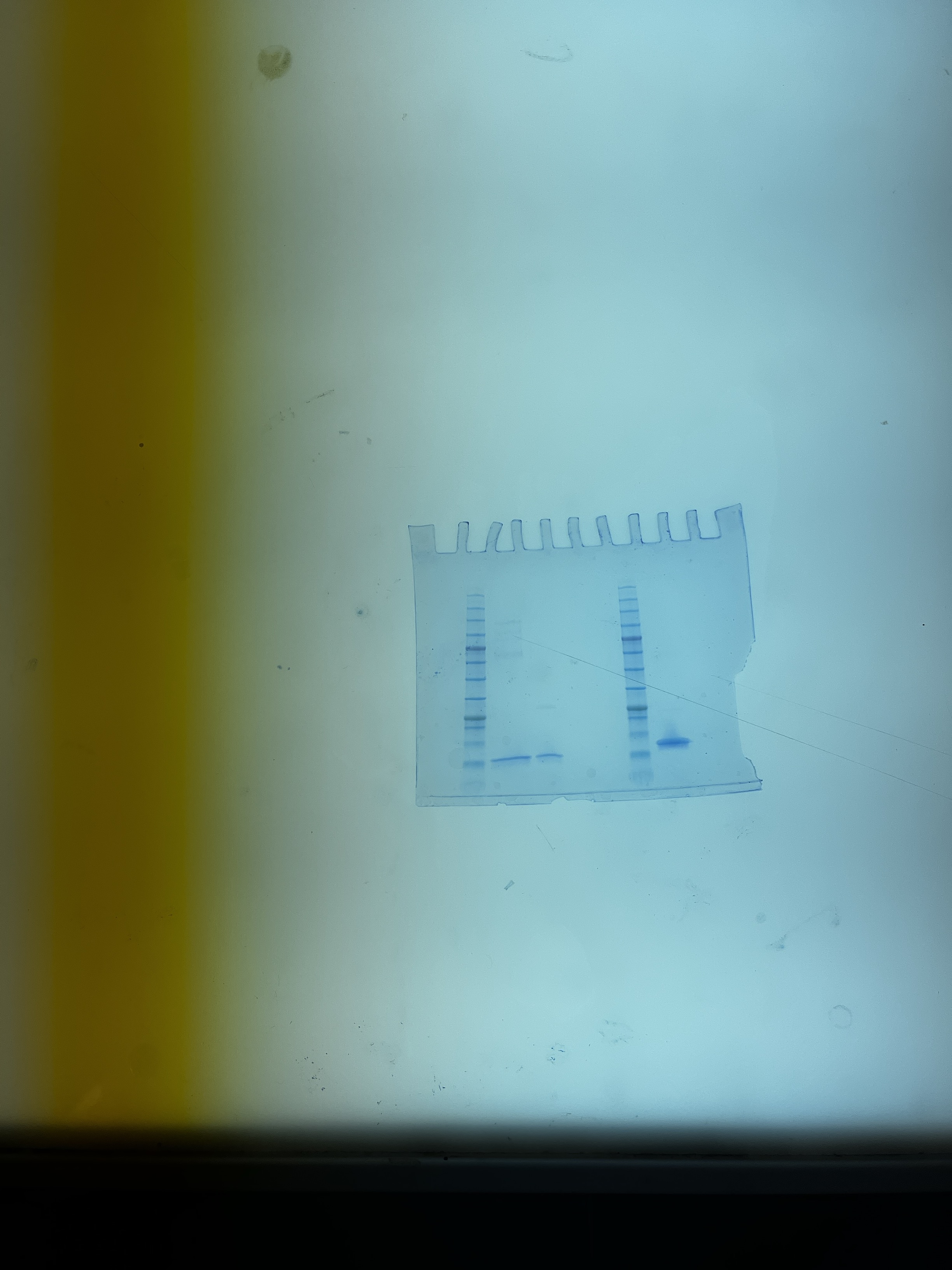

Supplement: Supplement 1 [file media-1.zip › Figure 1 - source data 1A.png]

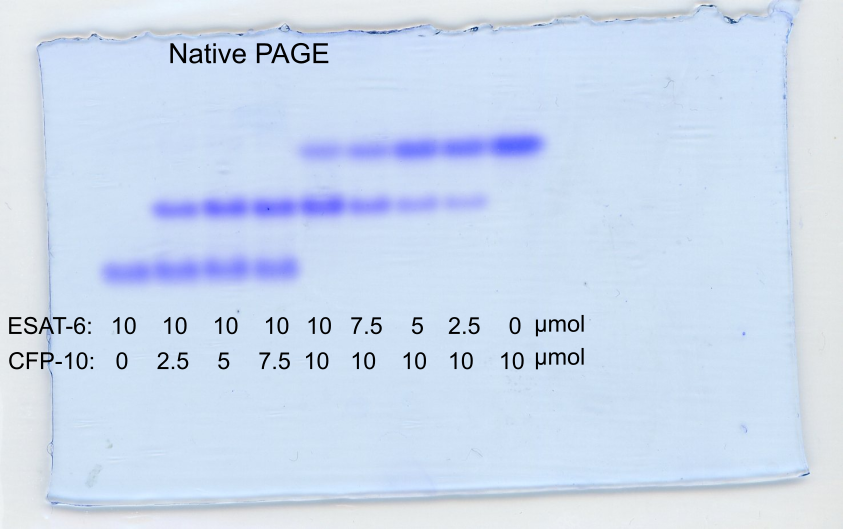

Supplement: Supplement 2 [file media-2.zip › Figure 2 - source data 1.png]

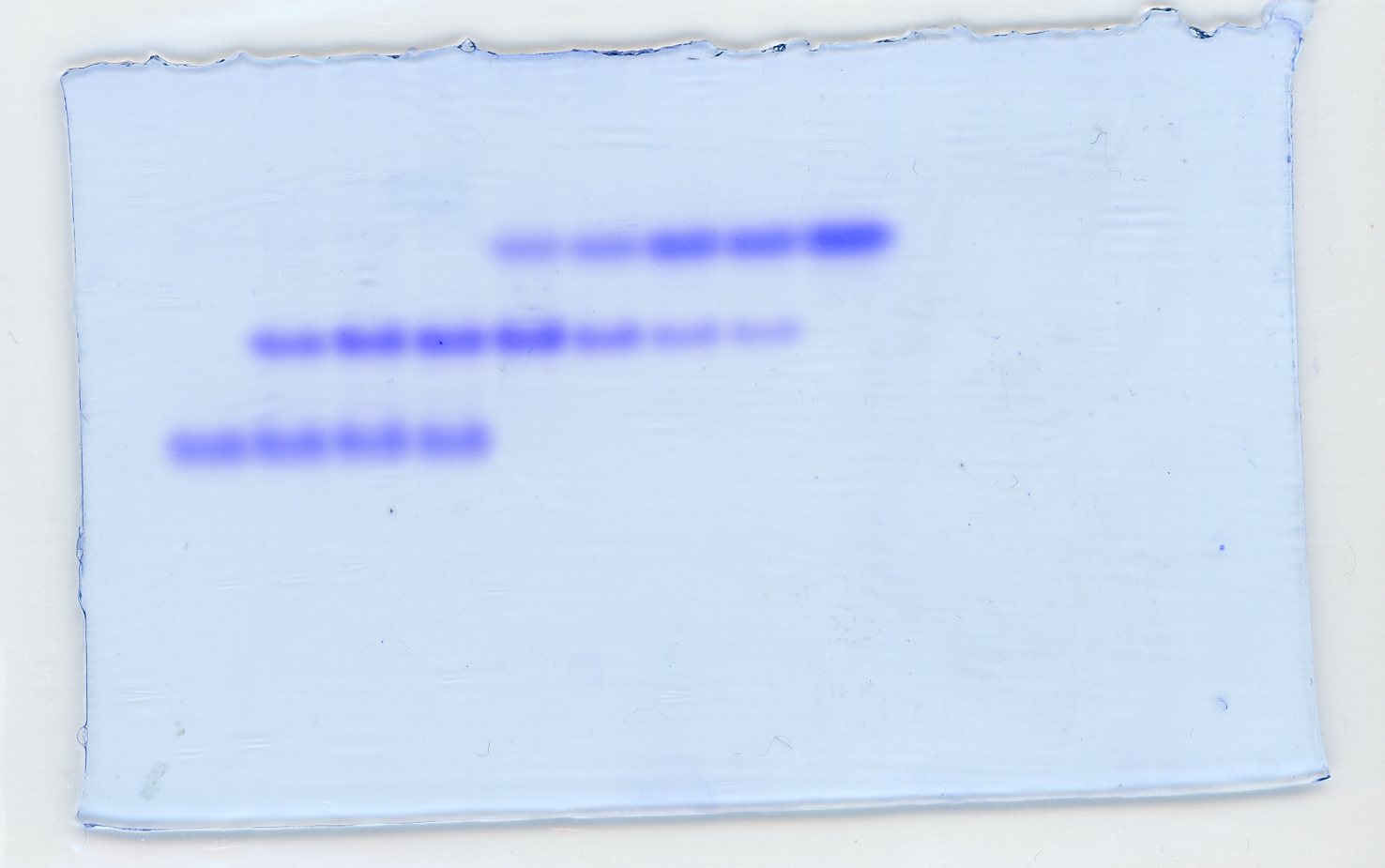

Supplement: Supplement 2 [file media-2.zip › Figure 2 - source data 1A.jpg]
