# Supplementary material for: ESAT-6 undergoes self-association at phagosomal pH and an ESAT-6 specific nanobody restricts M. tuberculosis growth in macrophages
Source: bioRxiv. 2024 Mar 19:2023.08.16.553641. Originally published 2023 Aug 17. Preprint. [Version 3] doi: 10.1101/2023.08.16.553641 (PMC10462100; doi:10.1101/2023.08.16.553641)
Supplement: Supplement 3 [file NIHPP2023.08.16.553641v3-supplement-3.pdf]

bioRxiv preprint doi: https://doi.org/10.1101/2023.08.16.553641; this version posted March 19, 2024. The copyright holder for this preprint (which was not certified by peer review) is the author/funder, who has granted bioRxiv a license to display the preprint in perpetuity. It is made available under aCC-BY 4.0 International license.

Figure 1 – figure supplement 1: Sequencing. (A) Sanger sequencing of pMRLB.7 with the ESAT-6 open reading frame (ORF) highlighted grey. (B) Sanger sequencing of pMRLB.46 with the CFP-10 ORF highlighted grey.

Figure 1 – figure supplement 2: Whole protein mass spectrometry (MS) of recombinant ESAT-6 and CFP-10. (A) Deconvoluted mass spectrum of recombinant ESAT-6 MS run. (B) Deconvoluted mass spectrum of recombinant CFP-10 MS run. (C) Computed mass of each protein compared with the measured masses. Fractional abundance combined the main peak with the harmonic artifact at twice the mass and gives an approximate relative proportion of each molecule in the sample. ESAT-6 is approximately 38% demethionylated while CFP-10 is fully demethionylated.

Figure 1 – source data 1: SDS-PAGE and Western blot. (A) Uncropped SDS-PAGE gel showing recombinant ESAT-6 and CFP-10 next to a BLUEstain 2 (Gold Bio) protein ladder and stained with Blazin' Blue (Goldbio) protein gel stain. (B) Uncropped western blot showing recombinant ESAT-6 and CFP-10 using a His-tag specific antibody.

bioRxiv preprint doi: https://doi.org/10.1101/2023.08.16.553641; this version posted March 19, 2024. The copyright holder for this preprint (which was not certified by peer review) is the author/funder, who has granted bioRxiv a license to display the preprint in perpetuity. It is made available under aCC-BY 4.0 International license.

Figure 2 – figure supplement 1: Hemolysis by recombinant ESAT-6. (A) Sheep red blood cell hemolysis assay carried out with purified ESAT-6 in citrate buffer at various pH values. Percent hemolysis was calculated by subtracting buffer background and dividing by Triton-X100 detergent lysed samples.

Figure 2 – source data 1: Native PAGE. (A) Native PAGE gel showing pure ESAT-6 on the left, pure CFP-10 on the right, and mixtures of both in the indicated ratios. The gel was stained with Blazin' Blue (Goldbio) protein gel stain.

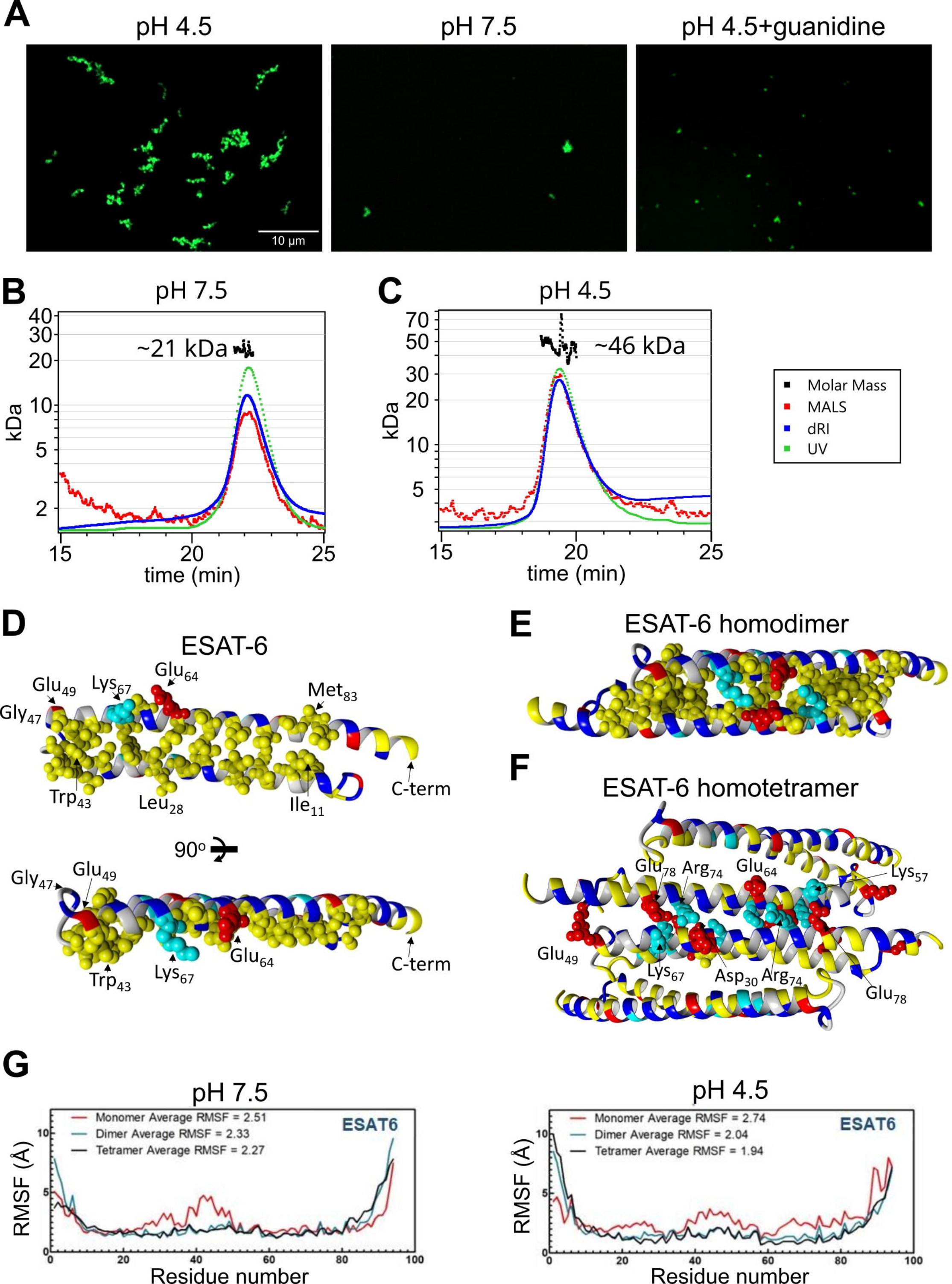
A
pH 4.5
pH 7.5
pH 4.5+guanidine
10 µm
B
pH 7.5
~21 kDa
kDa
time (min)
C
pH 4.5
~46 kDa
kDa
time (min)
Molar Mass
MALS
dRI
UV
D
ESAT-6
Glu49
Lys67
Glu64
Met83
Gly47
Trp43
Leu28
Ile11
C-term
90°
E
ESAT-6 homodimer
F
ESAT-6 homotetramer
Glu78
Arg74
Glu64
Lys57
Glu49
Lys67
Asp30
Arg74
Glu78
G
pH 7.5
Monomer Average RMSF = 2.51
Dimer Average RMSF = 2.33
Tetramer Average RMSF = 2.27
ESAT6
RMSF (Å)
Residue number
pH 4.5
Monomer Average RMSF = 2.74
Dimer Average RMSF = 2.04
Tetramer Average RMSF = 1.94
ESAT6
RMSF (Å)
Residue number

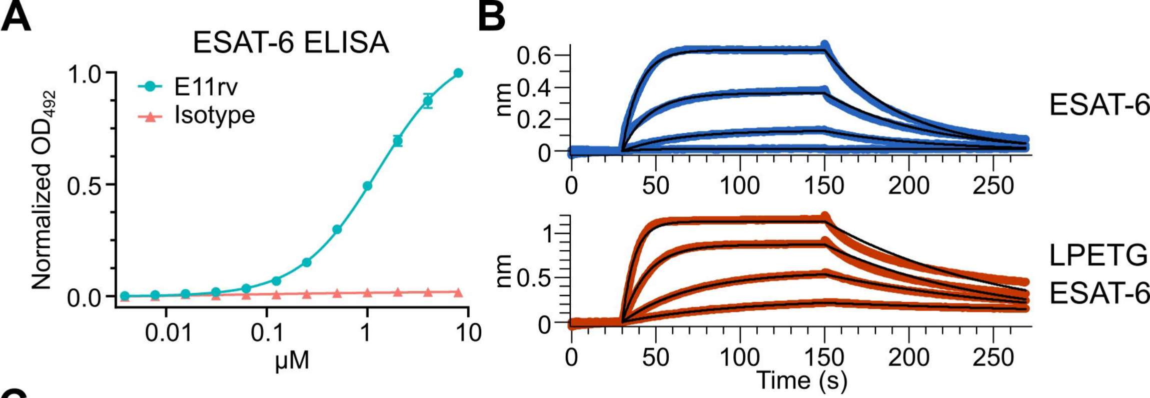

C

Attached	Soluble	pH	K_D (nM)	k_{ON} ($M^{-1}s^{-1}$)	k_{OFF} (s^{-1})
E11rv	ESAT-6	7.5	331	$5.5x10^4$	$1.8x10^{-2}$
E11rv	ESAT-6 LPETG	7.5	376	$1.9x10^4$	$7.0x10^{-3}$

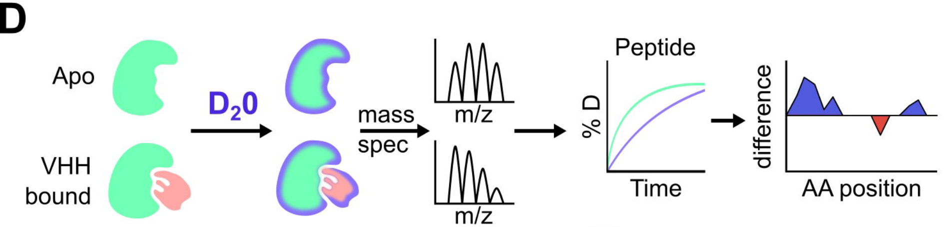

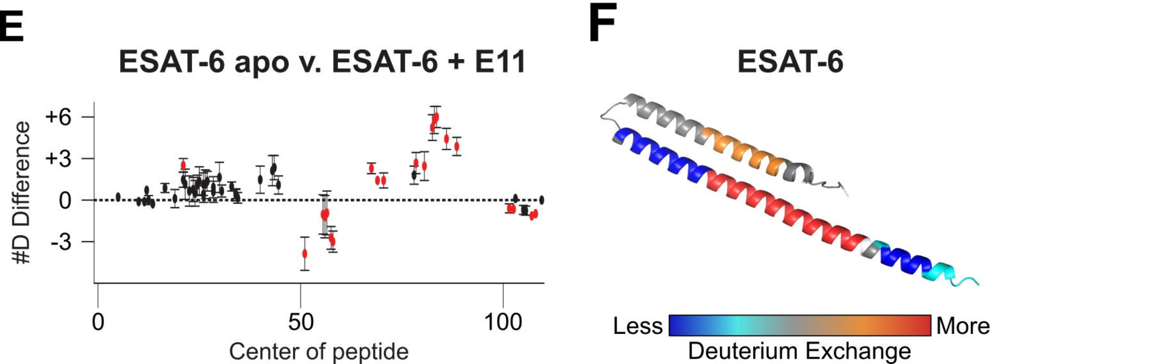

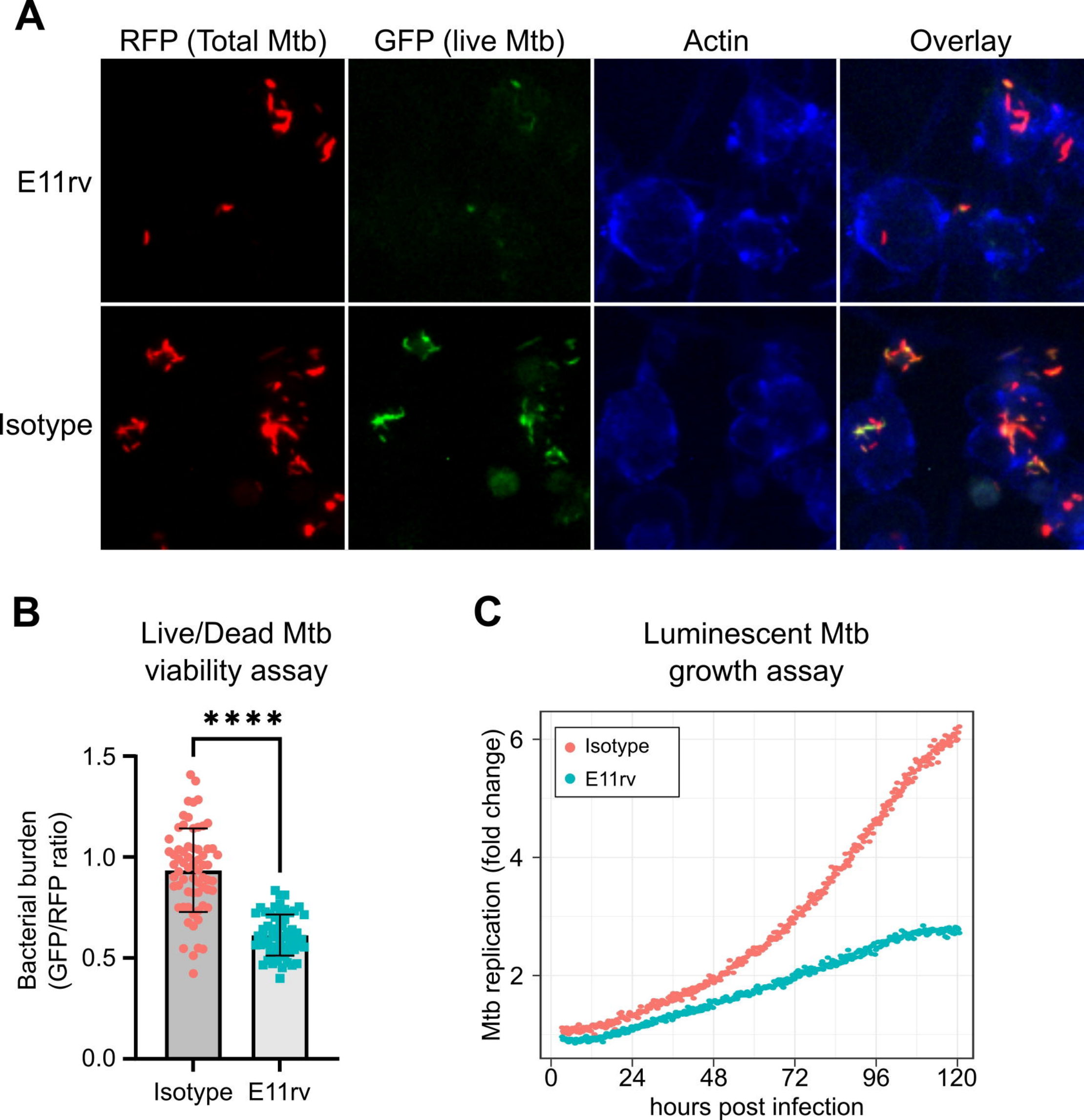
A
RFP (Total Mtb)
GFP (live Mtb)
Actin
Overlay
E11rv
Isotype
B
Live/Dead Mtb viability assay
****
Bacterial burden (GFP/RFP ratio)
0.0
0.5
1.0
1.5
Isotype
E11rv
C
Luminescent Mtb growth assay
Isotype
E11rv
Mtb replication (fold change)
2
4
6
0
24
48
72
96
120
hours post infection
