# Supplementary material for: ESAT-6 undergoes self-association at phagosomal pH and an ESAT-6 specific nanobody restricts M. tuberculosis growth in macrophages
Source: bioRxiv. 2023 Aug 17:2023.08.16.553641. Preprint. [Version 1] doi: 10.1101/2023.08.16.553641 (PMC10462100; doi:10.1101/2023.08.16.553641)
Supplement: Supplementary file 1 [file NIHPP2023.08.16.553641v1-supplement-1.pdf]

bioRxiv preprint doi: https://doi.org/10.1101/2023.08.16.553641; this version posted August 17, 2023. The copyright holder for this preprint (which was not certified by peer review) is the author/funder, who has granted bioRxiv a license to display the preprint in perpetuity. It is made available under aCC-BY 4.0 International license.

A

pMRLB.7 (ESAT-6)

```
              10        20        30        40        50        60
   1 CNNNNNNNCNNNNNANANTTTGTTTACTTTAAGAAGGAGATATACATATGACAGAGCAGC
  61 AGTGGAATTTCGCGGGTATCGAGGCCGCGGCAAGCGCAATCCAGGGAAATGTCACGTCCA
 121 TTCATTCCCTCCTTGACGAGGGGAAGCAGTCCCTGACCAAGCTCGCAGCGGCCTGGGGCG
 181 GTAGCGGTTCGGAGGCGTACCAGGGTGTCCAGCAAAAATGGGACGCCACGGCTACCGAGC
 241 TGAACAACGCGCTGCAGAACCTGGCGCGGACGATCAGCGAAGCCGGTCAGGCAATGGCTT
 301 CGACCGAAGGCAACGTCACTGGGATGTTCGCAGAGCTCATCAAGCTTATCGATACCGTCG
 361 ACCTCGAGCACCACCACCACCACCACTGAGATCCGGCTGCTAACAAAGCCCGAAAGGAAG
 421 CTGAGTTGGCTGCTGCCACCGCTGAGCAATAACTAGCATAACCCCTTGGGGCCTCTAAAC
 481 GGGTCTTGAGGGGTTTTTTGCTGAAAGGAGGAACTATATCCGGATTGGCGAATGGGACGC
 541 GCCCTGTAGCGGCGCATTAAGCGCGGCGGGTGTGGTGGTTACGCGCAGCGTGACCGCTAC
 601 ACTTGCCAGCGCCCTAGCGCCCGCTCCTTTCGCTTTCTTCCCTTCCTTTCTCGCCACGTT
 661 CGCCGGCTTTCCCCGTCAAGCTCTAAATCGGGGGCTCCCTTTAGGGTTCCGATTTAGTGC
 721 TTTACGGCACCTCGACCCCAAAAAACTTGATTANGGTGATGGTTCACGTAGTGGGCCATC
 781 GCCCTGATAGACGGTTTTTCGCCCTTTGACGTTGGAGTCCACGTTCTTTAATAGTGGACT
 841 CTTGTTCCAAACTGGNACAACACTCAACCCTATCTCGGTCTATTCTTTTGATTTATNANG
 901 NTTTTGCCGATTTCGGCCTATTGGNTAAAAATGAGCTGATTTAACAAAATTTAACGCGAA
 961 TTTANNNAATANTAACGCTTACATTTANNNGCACTTTTCGGGGAAATGTGCGNGACCCTN
1021 NTTGTTATTTTTCNAANACNTCANTATGNANCNCTCATGANANATANCNTGANAANGNTC
1081 ANAATNTNAAANNNNNNNATNANNATCANATTTCNNNNCNCCTATNCCNTTTNNNNGNNT
1141 TTNNNTNCNNNTTTNNNN
```

B

pMRLB.46 (CFP-10)

```
              10        20        30        40        50        60
   1 NNNNNNNNNNNNNNNNNNNNNNNNTTTTGTTTAACTTTAAGAAGGAGATATACATATGGC
  61 AGAGATGAAGACCGATGCCGCTACCCTCGCGCAGGAGGCAGGTAATTTCGAGCGGATCTC
 121 CGGCGACCTGAAAACCCAGATCGACCAGGTGGAGTCGACGGCAGGTTCGTTGCAGGGCCA
 181 GTGGCGCGGCGCGGCGGGGACGGCCGCCCAGGCCGCGGTGGTGCGCTTCCAAGAAGCAGC
 241 CAATAAGCAGAAGCAGGAACTCGACGAGATCTCGACGAATATTCGTCAGGCCGGCGTCCA
 301 ATACTCGAGGGCCGACGAGGAGCAGCAGCAGGCGCTGTCCTCGCAAATGGGCTTCAAGCT
 361 TGCGGCCGCACTCGAGCACCACCACCACCACCACTGAGATCCGGCTGCTAACAAAGCCCG
 421 AAAGGAAGCTGAGTTGGCTGCTGCCACCGCTGAGCAATAACTAGCATAACCCCTTGGGGC
 481 CTCTAAACGGGTCTTGAGGGGTTTTTTGCTGAAAGGAGGAACTATATCCGGATTGGCGAA
 541 TGGGACGCGCCCTGTAGCGGCGCATTAAGCGCGGCGGGTGTGGTGGTTACGCGCAGCGTG
 601 ACCGCTACACTTGCCAGCGCCCTAGCGCCCGCTCCTTTCGCTTTCTTCCCTTCCTTTCTC
 661 GCCACGTTCGCCGGCTTTCCCCGTCAAGCTCTAAATCGGGGGCTCCCTTTAGGGTTCCGA
 721 TTTAGTGCTTTACGGCACCTCGACCCCAAAAAACTTGATTAGGGTGATGGTTCACGTAGT
 781 GGGNCATCGCCCTGATAGACGGNTTTTCGCCCTTTGACGTTGGAGTCCACGTTCTTTAAT
 841 AGTGGACTCTTGTTCCAANCTGGAACAACACTCAACCCTATCTCGGTCTATTCTTTTGAT
 901 TTATANGGATTTTGCCGATTTCGGCCTATTGGTTAAAAAATGAGCTGANTTAACNAAAAT
 961 TTAACGCGAATTTTNACNAAATATTAACGCTTNNAANTTANNNGGCACTTTTCGGGGAAN
1021 NGNNNNCGGAACCCCTATTTGGTNNTTTTTTCTAAATACATNCAANNNGNANCCGCTCNT
1081 NANANATAACCNNGANNNNGCTTCANAANNTTGAAAAGGAANANNNNNAGTATTCNANAN
1141 NTCNNGNNNCCCNNNNNNNN
```

bioRxiv preprint doi: https://doi.org/10.1101/2023.08.16.553641; this version posted August 17, 2023. The copyright holder for this preprint (which was not certified by peer review) is the author/funder, who has granted bioRxiv a license to display the preprint in perpetuity. It is made available under aCC-BY 4.0 International license.

Supplemental Figure S1: Sequencing of. (A) Sanger sequencing of pMRLB.7 with the ESAT-6 open reading frame (ORF) highlighted grey. (B) Sanger sequencing of pMRLB.46 with the CFP-10 ORF highlighted grey.

bioRxiv preprint doi: https://doi.org/10.1101/2023.08.16.553641; this version posted August 17, 2023. The copyright holder for this preprint (which was not certified by peer review) is the author/funder, who has granted bioRxiv a license to display the preprint in perpetuity. It is made available under aCC-BY 4.0 International license.

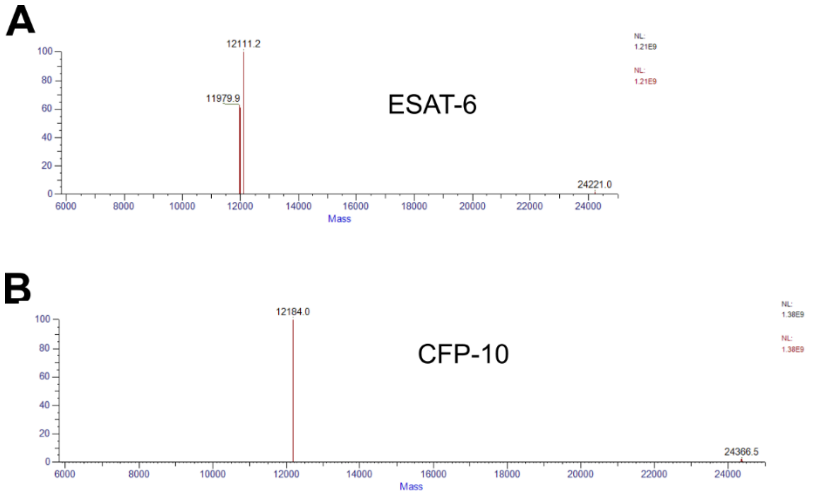

C

	Computed mass (Da)	Measured mass (Da)	Fractional abundance (%)
ESAT-6	12109.2	12111.2	62
ESAT-6ΔMet1	11978.0	11979.9	38
CFP-10	12313.4	ND	0
CFP-10ΔMet1	12182.2	12184.0	100

Supplemental Figure S2: Whole protein mass spectrometry (MS) of recombinant ESAT-6 and CFP-10. (A) Deconvoluted mass spectrum of recombinant ESAT-6 MS run. (B) Deconvoluted mass spectrum of recombinant CFP-10 MS run. (C) Computed mass of each protein compared with the measured masses. Fractional abundance combined the main peak with the harmonic artifact at twice the mass and gives an approximate relative proportion of each molecule in the sample. ESAT-6 is approximately 38% demethionylated while CFP-10 is fully demethionylated.

bioRxiv preprint doi: https://doi.org/10.1101/2023.08.16.553641; this version posted August 17, 2023. The copyright holder for this preprint (which was not certified by peer review) is the author/funder, who has granted bioRxiv a license to display the preprint in perpetuity. It is made available under aCC-BY 4.0 International license.

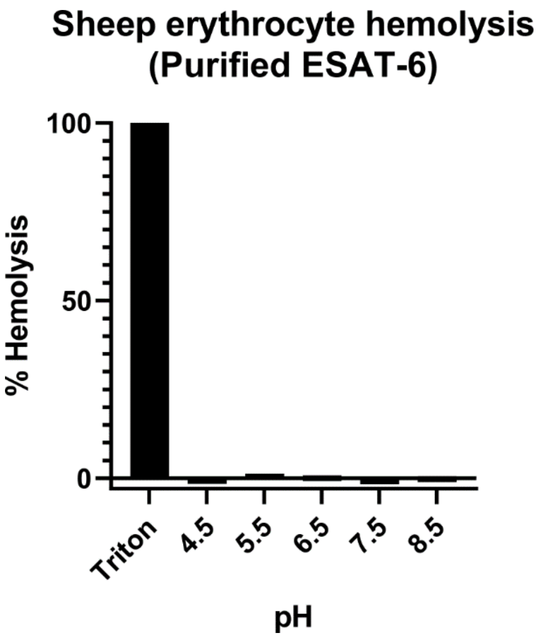

Supplemental Figure S3: Hemolysis by recombinant ESAT-6. (A) Sheep red blood cell hemolysis assay carried out with purified ESAT-6 in citrate buffer at various pH values. Percent hemolysis was calculated by subtracting buffer background and dividing by Triton-X100 detergent lysed samples.
